# Anti-CD52 antibody treatment depletes B cell aggregates in the central nervous system in a mouse model of multiple sclerosis

**DOI:** 10.1186/s12974-018-1263-9

**Published:** 2018-08-11

**Authors:** Micha Simon, Rojda Ipek, György A. Homola, Damiano M. Rovituso, Andrea Schampel, Christoph Kleinschnitz, Stefanie Kuerten

**Affiliations:** 1 Department of Anatomy and Cell Biology, University of Würzburg, Würzburg, Germany; 2 Department of Diagnostic and Interventional Neuroradiology, University Hospital Würzburg, Würzburg, Germany; 3 Department of Neurology, University Hospital Würzburg, Würzburg, Germany; 4 Department of Neurology, University Hospital Essen, Essen, Germany; 5 Institute of Anatomy and Cell Biology, Friedrich Alexander University Erlangen-Nürnberg (FAU), Krankenhausstr. 9, 91054 Erlangen, Bavaria Germany

**Keywords:** Alemtuzumab, B cells, CD52, CNS, EAE, MS

## Abstract

**Background:**

Multiple sclerosis (MS) is a chronic autoimmune disease of the central nervous system (CNS) for which several new treatment options were recently introduced. Among them is the monoclonal anti-CD52 antibody alemtuzumab that depletes mainly B cells and T cells in the immune periphery. Considering the ongoing controversy about the involvement of B cells and in particular the formation of B cell aggregates in the brains of progressive MS patients, an in-depth understanding of the effects of anti-CD52 antibody treatment on the B cell compartment in the CNS itself is desirable.

**Methods:**

We used myelin basic protein (MBP)-proteolipid protein (PLP)-induced experimental autoimmune encephalomyelitis (EAE) in C57BL/6 (B6) mice as B cell-dependent model of MS. Mice were treated intraperitoneally either at the peak of EAE or at 60 days after onset with 200 μg murine anti-CD52 vs. IgG2a isotype control antibody for five consecutive days. Disease was subsequently monitored for 10 days. The antigen-specific B cell/antibody response was measured by ELISPOT and ELISA. Effects on CNS infiltration and B cell aggregation were determined by immunohistochemistry. Neurodegeneration was evaluated by Luxol Fast Blue, SMI-32, and Olig2/APC staining as well as by electron microscopy and phosphorylated heavy neurofilament serum ELISA.

**Results:**

Treatment with anti-CD52 antibody attenuated EAE only when administered at the peak of disease. While there was no effect on the production of MP4-specific IgG, the treatment almost completely depleted CNS infiltrates and B cell aggregates even when given as late as 60 days after onset. On the ultrastructural level, we observed significantly less axonal damage in the spinal cord and cerebellum in chronic EAE after anti-CD52 treatment.

**Conclusion:**

Anti-CD52 treatment abrogated B cell infiltration and disrupted existing B cell aggregates in the CNS.

**Electronic supplementary material:**

The online version of this article (10.1186/s12974-018-1263-9) contains supplementary material, which is available to authorized users.

## Background

Multiple sclerosis (MS) is one of the most prevalent autoimmune disorders of the central nervous system (CNS) [1]. The disease is characterized by inflammation, demyelination, and axonal degeneration [2], eventually leading to severe and irreversible neurological deficits. The typical age of MS onset is between 20 and 40 years with a ratio of 2:1 female to male patients and a higher prevalence in the northern hemisphere. While the etiology of MS is not fully understood, a combination of genetic, environmental, and infectiological factors is assumed to be responsible for disease initiation and progression [1].

Traditionally, the autoimmune pathology of MS has been considered to be mainly T cell-driven [3], while more recent clinical observations as well as experimental data suggest a close interaction of T cells and B cells. Along these lines, depositions of immunoglobulins and of complement components have been found in active demyelinating brain lesions of MS patients [4], and oligoclonal bands (OCB) in the cerebrospinal fluid (CSF) of the majority of patients demonstrate local antibody production. Apart from antibody production, B cells function as highly potent antigen-presenting cells [5] particularly in the brain and are furthermore able to regulate ongoing inflammatory processes, e.g., by secreting pro- and/or anti-inflammatory cytokines. In addition, the overall clinical efficacy of B cell-depleting anti-CD20 antibody treatment and the therapeutic success of plasma exchange in a subset of MS patients with severe corticosteroid-resistant relapses further underlines the involvement of B cells in the immunopathology of MS [6, 7].

Alemtuzumab (Campath-1H) is a humanized anti-CD52 antibody [8], which was first approved for the treatment of patients with chronic lymphocytic leukemia. Alemtuzumab was later shown to be highly effective in patients with relapsing-remitting MS (RR-MS), lowering both the annual relapse rate and the sustained accumulation of disabilities in multiple clinical studies [9]. Improvement of pre-existing disabilities has also been reported. Cells of the acquired immune system, such as T cells and B cells, show high expression of CD52 molecules [10], hence being reduced drastically in numbers in the course of treatment, while cells of the innate system, such as natural killer cells and monocytes, are depleted to a lower extent due to lower CD52 expression. Plasma cells and hematopoietic stem cells lack CD52 and therefore remain unaffected. The absence of CD52 on hematological precursor cells enables maturation and repopulation after treatment, which appears to have a long-lasting tolerance-inducing effect in patients with MS, with more than 50% not requiring disease-modifying therapy 3 years after treatment [11]. This may be due to the slow repopulation of T cells with a relative expansion of CD4^+^CD25^+^CD127^low^ regulatory T cells [12–14] as well as a rapid repopulation of mostly immature B cells transiently exceeding baseline levels [15, 16]. While there is no doubt about the value of alemtuzumab for the treatment of RR-MS, further studies are needed to determine if it is equally effective in the case of progressive MS and also to determine whether there are patient subgroups for which individualized treatment would be appropriate.

To further study the immunological mechanisms of action of alemtuzumab, a human CD52 transgenic mouse strain was established [17]. In this mouse strain, both the distribution of CD52 molecules on leukocytes and the depletory capacity of alemtuzumab are comparable to that in humans [18]. However, for the ease of experimentation, a murine anti-CD52 antibody was subsequently established and used in experimental autoimmune encephalomyelitis (EAE) [19], which is the most common animal model of MS. Studies of anti-CD52 treatment in EAE have so far involved the myelin oligodendrocyte glycoprotein (MOG) peptide 35-55 [19–21] and the rMOG:1-121 model in C57BL/6 (B6) mice, as well as the relapsing-remitting proteolipid protein (PLP) peptide 13-151 model in SJL mice [19]. In all of these models, mice were treated on five consecutive days in an early disease stage, which resulted in the depletion of T cells and B cells comparable to MS patients treated with alemtuzumab. Anti-CD52-mediated lymphocyte depletion significantly attenuated clinical EAE, altered peripheral cytokine production, and reduced CNS inflammation, demyelination, and axonal damage. In addition, elevated levels of CD39^+^ regulatory T cells were observed in the gut-associated lymphatic tissue [20]. Since none of the aforementioned studies has used a B cell- and antibody-dependent model, the impact of anti-CD52 treatment on the peripheral vs. central B cell compartment in EAE still remains to be investigated. In addition, the antibody was previously given already with the onset of EAE when scores had not yet reached their maximal severity. Thus, it is unclear whether anti-CD52 antibody treatment is equally effective in more severe forms of EAE and in the chronic stage of the disease. To address these questions, we have employed the MP4-induced EAE model, in which mice are immunized with a fusion protein of myelin basic protein (MBP) and proteolipid protein (PLP) [22–24], and in which both pathogenic B cells and antibodies are induced. Our data demonstrate differential clinical effects of anti-CD52 treatment in acute and chronic EAE. In addition, this study is the first to show that treatment was highly effective in targeting both peripheral and central B cells, including those B cells that had already formed aggregates in the CNS. The disruption of these B cell aggregates was accompanied by a significant decrease in axonal pathology, even in late stages of the disease.

## Methods

### Mice

Six-week-old female B6 were purchased from Envigo (The Netherlands) and maintained under specific pathogen-free conditions at the animal facility of the Zentrum für Mund- und Kiefergesundheit at the University of Würzburg. The mice had free access to a standard rodent diet (Altromin Spezialfutter GmbH & Co. KG, Lage, Germany) and autoclaved water. Food was softened and kept at ground level for mice that displayed paralytic symptoms to ensure a sufficient diet. All animal experiments were performed according to protocols that were approved by the Regierung von Unterfranken (approval number 91/14) and complied with the German Law on the Protection of Animals and the “Principles of laboratory animal care” (NIH publication no. 86–23, revised 1985).

### EAE induction and clinical score

For EAE induction, incomplete Freund’s adjuvants was prepared by mixing paraffin oil (EM Science, Gibbstown, NJ, USA) and mannid monooleate (Sigma-Aldrich, St. Louis, USA) at a 9:1 ratio. To obtain complete Freund’s adjuvants (CFA), 5 mg/ml *Mycobacterium tuberculosis* H37 Ra (Difco Laboratories, Franklin Lakes, NJ, USA) was added. Each mouse was immunized subcutaneously into both sides of the flank with a total dose of 200 μg MP4 (Alexion Pharmaceuticals, Cheshire, CT, USA), emulsified in a total volume of 200 μl CFA. In addition, mice received 200 ng pertussis toxin (List Biological Laboratories, Hornby, ONT, Canada) by intraperitoneal injection at the day of immunization and 48 h later. Mice were evaluated daily to record onset and progression of clinical symptoms based on the standard EAE scoring system: (0) no disease, (1) floppy tail, (2) hind limb weakness, (3) full hind limb paralysis, (4) quadriplegia, and (5) death. Increments of 0.5 were used to account for clinical deficits in between the defined hallmarks.

### Treatment

Mice were treated either with a 200 μg (10 mg/kg body weight) anti-mCD52 antibody, obtained from Sanofi Genzyme (Cambridge, MA, USA), or with a murine IgG2a isotype control antibody (InVivo, Henningdorf, Germany) for five consecutive days. Treatment was given by intraperitoneal injection, and mice were subsequently monitored daily for at least 10 days to determine the treatment effect. Mice were treated either at the peak of EAE (“acute EAE”) or at ~ 60 days after EAE onset (“chronic EAE”). For randomization purposes, each mouse in each cohort was assigned to one of the two treatment groups in an alternating fashion once the mouse had developed EAE. Yet, the extent of CNS inflammation was almost exclusively dependent on the EAE score, rather than on the disease duration after onset. Hence, slight variations of the initial randomization strategy occurred since the two groups were score-matched at the beginning of the treatment (Table 1).

**Table 1:** Clinical parameters of EAE in mice treated with IgG2a isotype control or anti-mCD52 antibody

	EAE onset (days after immunization)	Score at treatment onset	Disease duration before treatment	Final score	Score difference
Treatment at the peak of EAE					
Isotype					
*n* = 5	15.40 ± 1.40	2.75 ± 0.08	5.20 ± 1.83	2.15 ± 0.22	0.60 ± 0.15
Anti-mCD52					
*n* = 4	20.25 ± 1.11	2.94 ± 0.36	2.75 ± 1.11	1.50 ± 0.54	1.44 ± 0.21
*p* value	0.02	0.83	0.37	0.32	0.02
Treatment after ~ 60 days of EAE					
Isotype					
*n* = 14	16.29 ± 0.85	2.61 ± 0.09	61.57 ± 1.27	2.43 ± 0.15	0.18 ± 0.10
Anti-mCD52					
*n* = 18	15.28 ± 0.85	2.57 ± 0.11	61.72 ± 1.20	2.47 ± 0.13	0.10 ± 0.07
*p* value	0.29	0.59	0.95	0.85	0.47

All data are shown as mean values ± SEM. Mann-Whitney test was used to determine statistical significance

### Tissue sampling and preparation

Blood samples for flow cytometry were taken from the tail vein 1 day before sacrifice. Mice were sacrificed with CO_2_ before a non-perfused draining inguinal lymph node was collected for ELISPOT and blood was drawn from the inferior vena cava for ELISA analysis. Mice were then perfused with 4% paraformaldehyde (PFA) (Roth, Karlsruhe, Germany) in 0.01 M phosphate-buffered saline (PBS) (pH 7.4) for immunohistochemistry (IHC) or with 4% PFA/4% glutaraldehyde (GA) (Roth, Karlsruhe, Germany) in 0.01 M PBS (pH 7.4) for electron microscopy. The cerebellum, lumbar spinal cord, and the other draining inguinal lymph nodes were carefully dissected and post-fixed overnight at 4 °C under slight agitation. While samples for IHC were embedded in paraffin, samples for electron microscopy were embedded in Epon.

### Flow cytometry

Blood was collected from the tail vein into heparinized tubes (Liquemin N25000, Roche, Basel, Switzerland). The blood was diluted 1:9 with PBS (total volume 1 ml), and red blood cells were lysed using an ammonium chloride-based lysis buffer. All samples were washed in PBS, transferred to cell culture plates (Greiner bio-one, Kremsmünster, Austria), and stained for 30 min with Fixable Viability Stain 450 (BD Biosciences, San Jose, CA, USA). Staining of T and B cells was performed with rat anti-mouse PerCP-Cy5.5-labeled CD4 (BD Biosciences) and APC-labeled rat anti-mouse CD19 antibody (BioLegend, San Diego, CA, USA) at 4 °C for 30 min. Samples were measured using a BD FACSCanto II (BD Biosciences) with BD FACS Diva software (version 6.1.3) at a flowrate of 1000–2000 events/min until at least 15,000 events were recorded. Further analysis was performed with FlowJo® (version 10.0.6, Tree Star Inc., Ashland, USA). Only viable single cells were included in the analysis. A lymphocyte gate on the FSC-H (forward scatter height)/FSC-A (forward scatter area) profile was set. B cells were characterized as CD19^+^CD4^−^ and T cells as CD19^−^CD4^+^ applying a CD4/CD19 bivariate gate. Gates were first set identically for all samples and adjusted individually according to unstained samples. The overall gating strategy is demonstrated in Additional file 1.

### ELISPOT analysis

MultiScreen HTS 96-well 0.45-μm ELISPOT plates (Merck-Millipore, Darmstadt, Germany) were coated overnight at 4 °C with 100 μl per well of either 10 μg/ml MP4 (Alexion Pharmaceuticals) or 15 μg/ml anti-mouse IgG (MabTech, Nacka Strand, Sweden) in sterile PBS (Sigma-Aldrich) or with sterile PBS only for negative controls. Plates were blocked with 10% fetal bovine serum (FBS; Gibco, Thermo Fisher Scientific, Waltham, MA, USA) in sterile PBS at room temperature for 2 h. Non-perfused inguinal lymph nodes were dissected, and the suspension was passed through a 70-μm cell strainer (Falcon, Corning, NY, USA). Cells were washed in RPMI-1640 medium before counting them with 0.2% trypan blue (Roth). Samples were resuspended in HL-1 (Lonza, Basel, Switzerland) containing 1% l-glutamine (Sigma-Aldrich) and 1% penicillin/streptomycin (Sigma-Aldrich) and plated at a concentration of 10^6^ cells/well. Plates were incubated at 37 °C and 7% CO_2_ for 24 h. Goat anti-mouse IgG served as secondary antibody (Dako, Glostrop, Denmark) diluted 1:2000 in 0.5% FBS/PBS and was incubated overnight at 4 °C. Afterwards, streptavidin-alkaline phosphatase (1:800) in 0.5% FBS/PBS was incubated at room temperature for 2 h before Vector Blue substrate kit (Vector Laboratories, Burlingame, CA, USA) was applied according to the manufacturer’s instructions. Scanning and image analysis of the plates was performed using an ImmunoSpot Series 6 UV Analyzer (CTL-Europe, Bonn, Germany).

### MP4-specific antibody ELISA

ELISA plates (Nunclon™ Delta Surface™ 96 MicroWell, Nalge Nunc International, Rochester, NY, USA) were coated overnight at 4 °C with 100 μl of 3 μg/ml MP4 in PBS. The plates were blocked with 1% milk powder (MP; Herler Bio Magermilchpulver, Heirler-Cenovis GmBH) in PBS/0.05% Tween (Biochemica, Billingham, UK) at room temperature for 2 h. The serum samples were diluted 1:1000 in 1% MP/0.05% Tween in PBS and incubated at 4 °C overnight. Negative control wells contained 1%MP/0.05% Tween in PBS. Biotinylated anti-mouse IgG (eBioscience, Waltham, MA, USA) was diluted 1:800 in 0.1% MP/PBS and incubated overnight at 4 °C. Strepavidin-horseradish peroxidase (BD Biosciences) was diluted 1:1000 in 0.1% MP/PBS and added for 2 h before 100 μl tetrametyhylbenzidin substrate (eBioscience) was used for development. The reaction was stopped with 0.16 M sulfuric acid and analyzed at 450 nm using a Perkin Elmer (Waltham, MA, USA) Victor^3^ 1420 Multilabel Counter with Wallac 1420 software version 3.00 revision 5.

### IHC for CD3^+^ T cells and B220^+^ B cells

In MP4-induced EAE, a focus of B cell infiltration is observed in the cerebellum [25]. To this end, paraffin-embedded cerebella were cut into 4–5-μm-thick serial sections covering the entire tissue. Every seventh (acute treatment group) or ninth (chronic treatment group) section was stained with hematoxylin/eosin in order to screen for the presence of inflammatory infiltrates. To this end, *n* = 12 sections were analyzed per mouse. In case of infiltration, the adjacent section was stained for CD3^+^ T cells and B220^+^ B cells by IHC. The diaminobenzidine (DAB)/Vector Blue staining protocol consisted of the following steps: paraffin-embedded sections were rehydrated and endogenous peroxidase was blocked. After citrate buffer-mediated epitope retrieval and protein blocking, the primary antibodies rabbit anti-mouse CD3 (1:200; Abcam, Cambridge, UK) and rat anti-mouse B220 (1:500; eBioscience) were incubated in 1% normal goat serum (NGS; Sigma-Aldrich)/PBS at 4 °C overnight. Inguinal lymph node sections (5 μm thick) served as positive controls. Biotinylated anti-rabbit and anti-rat antibodies (both Vector Laboratories; diluted 1:250 in PBS) were used as secondary antibodies and incubated at room temperature for 1 h. For CD3 detection, the sections were incubated with rabbit-peroxidase-anti-peroxidase complex (diluted 1:250 in PBS) for 30 min in the dark. The sections were developed with a horseradish peroxidase-based VECTASTAIN ABC kit (Vector Laboratories) and monitored under microscopic control in a solution containing nickel sulfate, glucose, ammonium chloride, DAB, and glucose oxidase. For B220 staining, alkaline phosphatase-conjugated streptavidin and Vector Blue substrate kit (Vector Laboratories) were used. The sections were examined with a Leica DM 2000 LED microscope equipped with a Leica MC150HD digital camera and Leica Application Suite 4.40 Build 454 software (Leica, Wetzlar, Germany). Hematoxylin/eosin-stained sections were used to determine the parenchymal area of the cerebella by ImageJ 2.0.0.-rc-54/1.51b software (National Institutes of Health, USA). All infiltrates were further categorized into non-B cell infiltrates, diffuse B cell infiltrates, and B cell aggregates. Since anti-CD52 treatment completely abrogated the formation of B cell aggregates or disrupted these structures during chronic EAE, staining for lymphoid neogenesis was not performed.

### Assessment of demyelination and axonal damage by Luxol Fast Blue (LFB) and SMI-32 IHC staining

The spinal cords and cerebella of the chronic EAE cohort were cut into serial sections of 4 μm. Tissue was stained with LFB to detect demyelination (*n* = 12 sections per mouse) and with anti-SMI-32 antibody to detect axonal damage (*n* = 4 sections per mouse). The DAB staining protocol for SMI-32 consisted of the following steps: paraffin-embedded sections were rehydrated and endogenous peroxidase was blocked. After citrate buffer-mediated epitope retrieval and protein blocking, staining was performed with the M.O.M kit according to the manufacturer’s instructions (Vector Laboratories). The primary antibody was used at 1:500 dilution (Covance, Princton, NJ, USA). The sections were developed with a horseradish peroxidase-based VECTASTAIN ABC kit (Vector Laboratories) and monitored under microscopic control in a solution containing nickel sulfate, glucose, ammonium chloride, DAB, and glucose oxidase. For LFB staining, slides were also deparaffinized and subsequently stained with a 0.2% solution of Solvent Blue 38 (Sigma-Aldrich) in 96% ethanol at 60 °C overnight. The process was stopped in 50% ethanol, followed by distilled water. To distinguish between myelinated and non-myelinated areas, the slides were stored in 0.05% lithium carbonate solution for 30 s before removing the dye for 30 s in 75% ethanol and stopping the reaction in distilled water. Differentiation was repeated until gray and white matter were clearly distinguishable. All sections were examined with a Leica DM 2000 LED microscope. Analysis and quantification were done with ImageJ 2.0.0.-rc-54/1.51b (National Institutes of Health). Total white matter area and the demyelinated regions within the white matter were measured, and SMI-32^+^ axons were counted.

### Olig2^+^APC^+^ IHC

In the chronic EAE cohort, 4-μm-thick paraffin-embedded sections of *n* = 3 lumbar spinal cords and cerebella were deparaffinized and rehydrated before citrate buffer-based antigen retrieval was performed. Sections were permeabilized for 10 min in 0.25 Triton X/PBS before blocking in 5% NGS/PBS at room temperature for 1 h. Rabbit anti-mouse Olig2 antibody (1:500; Merck Milipore) was incubated at 4 °C overnight. The following day, sections were incubated with biotinylated goat anti-rabbit antibody (1:250; Vector Laboratories) in PBS and with DAPI (1:5000; Roche) at room temperature for 30 min. To perform double staining, the sections were again blocked in 5% NGS/PBS at room temperature for 1 h before incubation with APC primary antibody (Calbiochem, USA) diluted 1:100 in 1% NGS/PBS at room temperature for 2 h. Biotinylated goat anti-mouse IgG (Vector Laboratories) was subsequently applied at a dilution of 1:250 in 1% NGS/PBS at room temperature for 30 min before incubation with NeutrAvidin DyLight 550 (Thermo Scientific). Sections were coverslipped with Mowiol 4-88 (Roth). For analysis, six to eight images of each segment were acquired using a Keyence BZ-9000 fluorescence microscope (Osaka, Japan). Olig2^+^APC^+^ cells were counted and white matter area measurements were performed using ImageJ 2.0.0.-rc-54/1.51b software (National Institutes of Health).

### Phosphorylated neurofilament heavy (pNF-H) ELISA

pNF-H ELISA was performed with the sera of the same mice that were used for histological analysis. We used a commercially available ELISA kit from Merck according to the manufacturer’s instructions.

### Electron microscopy

For EM analysis, we obtained a standardized tissue block from the lumbar spinal cord of each mouse. We carefully prepared the whole spine and isolated the part that contained the spinal cord between thorax and the beginning of the cauda equina. Ultra-thin sections were then obtained from the top of each tissue block. For EM analysis of the cerebellum, the tissue of each mouse was cut into six similar blocks. These blocks corresponded to those areas that most likely contained B cell aggregates and thus neurodegeneration according to our previous experience. All six blocks were screened, and images were acquired from those blocks that were affected by neuroinflammation.

For embedding, samples were first washed with PBS and subsequently with 0.1 M cacodylate buffer (CACO, sodium dimethyl arsenate in aqua dest) (EMS, München, Germany) (pH 7.35) three times for 10 min each. Afterwards, samples were stored in 0.1 M CACO buffer at 4 °C under slight agitation. For epon embedding, samples were washed again with 0.1 M CACO buffer three times on a rotating wheel for 10 min each. Then, they were placed into 0.1 M CACO buffer containing 1% osmium tetroxide (OsO_4_) (EMS, München, Germany) at room temperature for 4 h. During that time, the tissue was protected from light and deposited on a rotator. The tissue was then washed with aqua bidest twice for 15 min and then for 15 min each in 30%, 50%, 70%, 80%, 90%, and 96% ethanol. After washing twice in 100% ethanol for 15 min each, the tissue was incubated in propylene oxide (EMS, München, Germany) twice for 30 min. The samples were then transferred into a 1:1 mixture of propylene oxide and epon at room temperature overnight and afterwards incubated with pure fresh epon for 2 h. Epon was changed again, and embedded tissue samples were polymerized at 60 °C for 48 h. Epon was prepared by mixing 26 g glycid ether (Serva, Heidelberg, Germany), 11 g dodecenyl succinic-acid anhydride (DDSA) (Serva, Heidelberg, Germany), 15 g methylacid anhydride (MNA) (Serva, Heidelberg, Germany), and 0.25 g 2-,4-,6-tris(dimethylaminomethyl)phenole (DMP) (Serva, Heidelberg, Germany). Embedded tissue was cut transversely at 1 μm on an ultra-microtome (Leica, Wetzlar, Germany, serial number 365952) for semi-thin analysis. Sections were dried at 60 °C for at least 20 min and rinsed in methylene blue (Merck, Darmstadt, Germany) for approximately 45 s. After sections had been washed and dried on a heating plate, they were covered in DEPEX (Serva, Heidelberg, Germany). Digital images were acquired using a Leica DM LB2 microscope, equipped with a Zeiss camera (AxioCam MRc) and Zeiss software (AxioVision 40 4.7). For EM analysis, tissue was cut into 65–85-nm-thick sections. Sections were stretched with chloroform (Roth, Karlsruhe, Germany) while floating in the water tub of the diamond knife (EMS, München, Germany). Afterwards they were carefully put on 100 mesh formvar-coated nickel grids (EMS, München, Germany). For contrast enhancement, the sections were stained with 2% uranyl acetate (Serva, Heidelberg, Germany) in 70% ethanol for 20 min and Reynold’s lead citrate solution (Plano, Wetzlar, Germany) for 7 min. The tissue was analyzed using a Zeiss AB912 LEO transmission electron microscope at 80-kV acceleration voltage. Digital images were acquired with an EM digital camera system (Tröndle sharp eye camera, Moorenweis, Germany) equipped with Zeiss software (Image SP × 64) at × 6300 magnification. At least 10 images per mouse and tissue were analyzed, which covered the areas of the spinal cord and cerebellar lesions.

### Statistical analysis

Mann-Whitney and one-way ANOVA tests were used for statistical analysis and computed using GraphPad Prism 6 (La Jolla, CA, USA). Statistical significance was set at *p* ≤ 0.05. Mean values and standard errors of the mean (SEM) are shown in the graphs.

## Results

### Anti-mCD52 antibody treatment attenuates acute but not chronic MP4-induced EAE

**Fig. 1:**
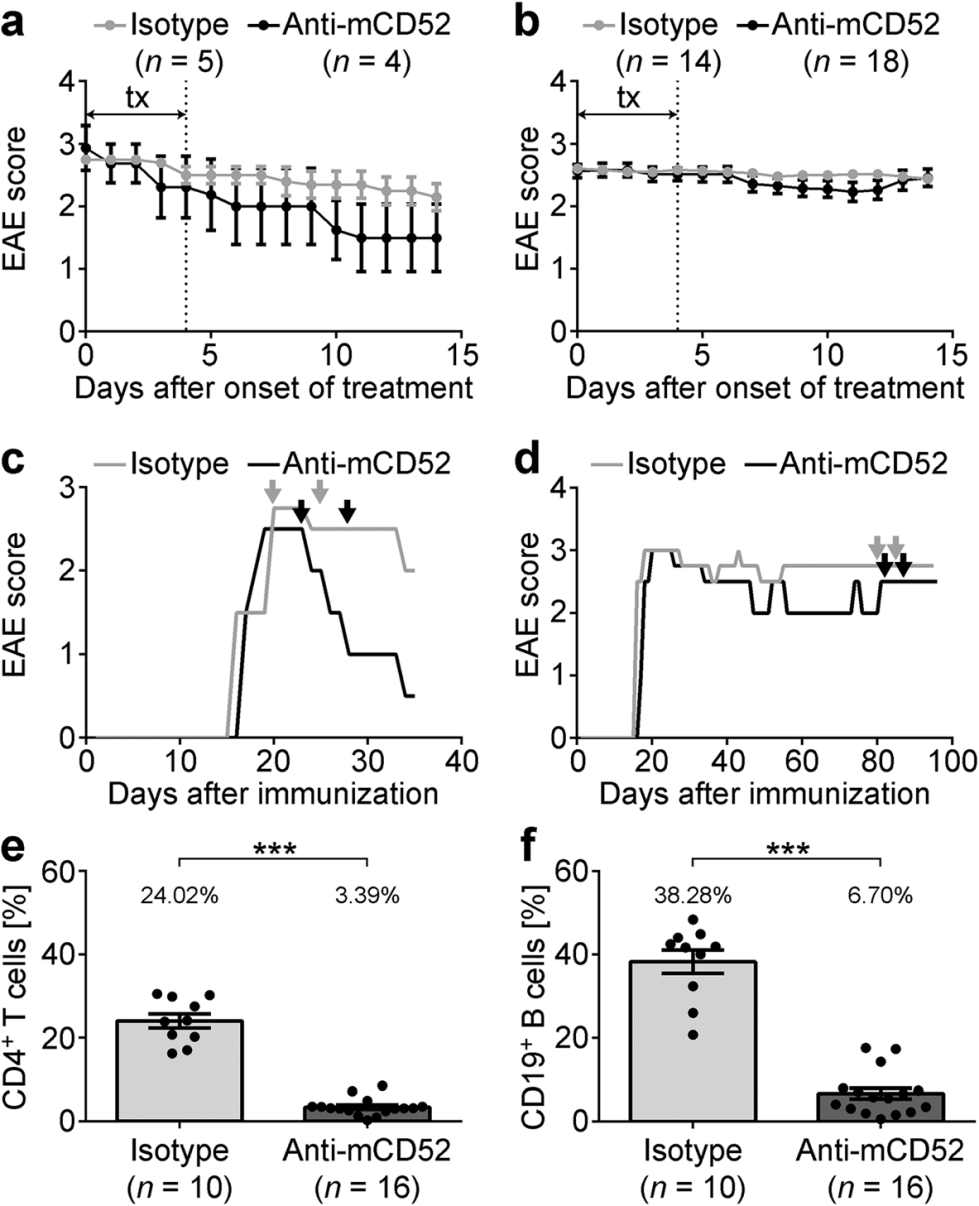
Disease course in mice treated with anti-mCD52 antibody in the acute or chronic stage of EAE. MP4-immunized mice were treated with 200 μg anti-mCD52 antibody or IgG2a isotype control for five consecutive days (tx) either **a** at the peak of the disease or **b** at ~ 60 days after EAE onset. **c**, **d** Representative disease course of individual animals treated during **c** acute or **d** chronic EAE. Arrows indicate the treatment period. **e**, **f** Flow cytometry of CD4^+^ T cells and CD19^+^ B cells in the blood after treatment with anti-mCD52 compared to vehicle. ***p < 0.001, Mann-Whitney test. Mean values ± SEM are shown. For EAE scores, statistics were calculated based on differences on a daily basis

MP4-immunized B6 mice received 200 μg of anti-mCD52 antibody or IgG2a isotype control by intraperitoneal injection on five consecutive days either at the peak of acute EAE or at ~ 60 days after EAE onset. The mean EAE onset of all treated mice was 16.12 ± 0.55 days after immunization. There was no significant difference in disease severity in anti-mCD52- and isotype control-treated mice at the beginning of treatment in both acute (*p* = 0.83) and chronic (*p* = 0.59) EAE (Fig. 1a–d, Additional file 2, Table 1). In acute EAE, anti-mCD52-treated mice displayed a significant decrease in disease severity 11.56 ± 0.18 days post-treatment, while the scores of isotype control-treated mice remained unaffected (Fig. 1a, Table 1; *p* = 0.02). In chronic EAE, anti-mCD52 treatment did not have any effect on the difference between pre- and post-treatment scores (Fig. 1b; Table 1; *p* = 0.47). In order to determine the extent of depletion of circulating lymphocytes after anti-mCD52 treatment, we performed flow cytometry on tail vein blood obtained 10.37 ± 0.18 days post-treatment and we determined the percentages of viable CD4^+^ T cells and CD19^+^ B cells. In the chronic cohort, CD4^+^ T cells showed a significant reduction from 24.02 ± 1.71% to 3.39 ± 0.52% (*p* < 0.001; Fig. 1e). The percentage of CD19^+^ B cells was significantly reduced to 6.70 ± 1.23% in anti-mCD52-treated mice compared to 38.28 ± 2.82% in the isotype control group (*p* < 0.001; Fig. 1f). In the acute EAE cohort, comparable results were observed for both T cells (compare 33.82 ± 3.68% to 0.92 ± 0.19%; *p* = 0.016) and B cells (compare 21.51 ± 4.41% to 3.22 ± 1.34%; *p* = 0.016).

### Anti-mCD52 treatment does not affect MP4-specific B cells and autoantibodies

**Fig. 2:**
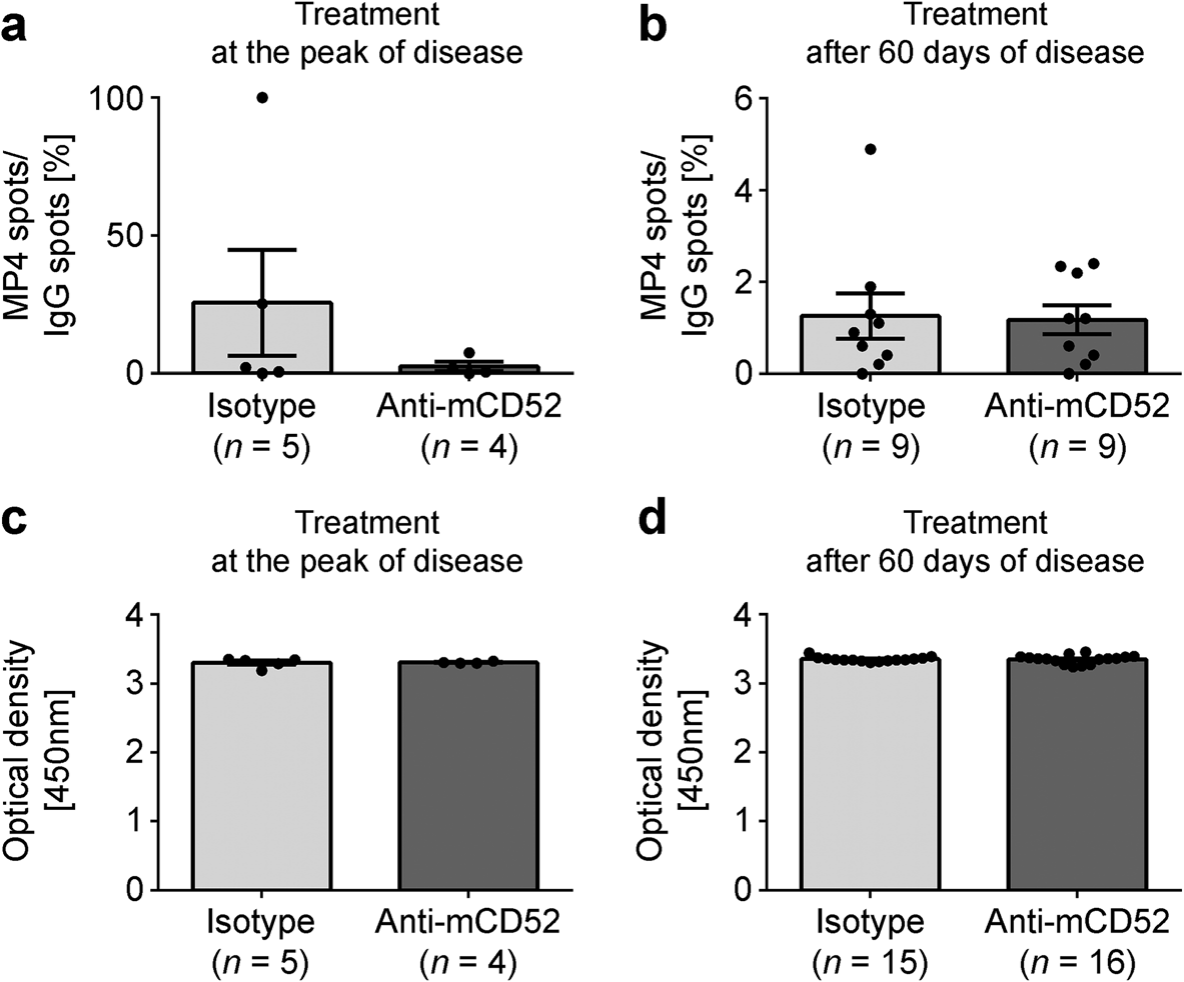
Measurements of MP4-specific B cells and antibodies. B cell ELISPOT was performed on draining inguinal lymph nodes of MP4-immunized mice to detect the number of MP4-specific B cell spots in relation to the number of total IgG spots during **a** acute or **b** chronic EAE. MP4-specific serum antibodies were measured by ELISA during **c** acute or **d** chronic treatment. Mice were treated with either 200 μg anti-mCD52 antibody or IgG2a isotype control for five consecutive days. Means ± SEM are shown

In order to determine the effect of anti-mCD52 treatment on peripheral MP4-specific B cells, we performed ELISPOT on draining lymph node cells (Fig. 2a, b) of both anti-mCD52- and isotype control-treated mice in the acute and chronic EAE cohort. Samples were collected 11.37 ± 0.18 days after treatment onset. As shown in Fig. 2, there was no difference between the numbers of MP4-specific antibody secreting B cells expressed as anti-MP4 IgG/total IgG ratio in anti-mCD52- vs. isotype control-treated animals (*p*_acute_ = 0.51; *p*_chronic_ = 0.81). There was also no difference between anti-MP4-specific IgG titers in the sera of both groups (*p*_acute_ = 0.73; *p*_chronic_ = 0.75) (Fig. 2c, d).

### Anti-mCD52 treatment significantly decreases immune cell infiltration in the cerebella of acute and chronic MP4-immunized mice

**Fig. 3:**
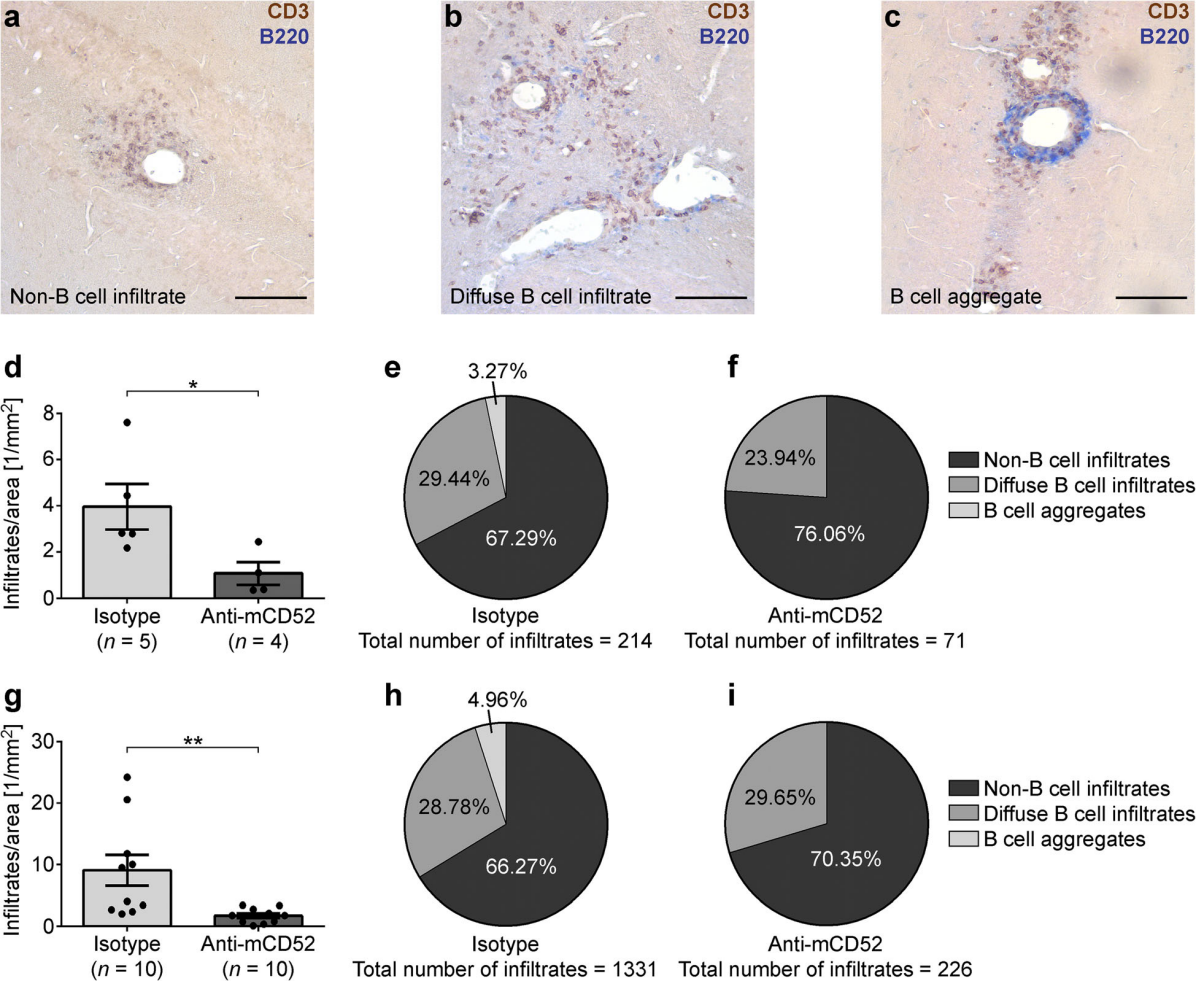
Histological analysis of B cell aggregates in the cerebella of MP4-immunized mice. **a**–**c** CD3/B220 double staining of representative cerebellar sections of a mouse treated with isotype control at the peak of disease. The panels demonstrate the different infiltrate categories. Scale bars represent 100 μm. **d**–**f** Quantification of the number of infiltrates per parenchymal area and distribution of infiltrate categories in mice treated either with anti-mCD52 antibody or isotype control at the peak of disease. **g**–**i** Quantification of the number of infiltrates per parenchymal area and distribution of infiltrate categories in mice treated ~ 60 days after disease onset. Graphs show means ± SEM. *p < 0.05, **p < 0.01, Mann-Whitney test

Since our results showed a reduction in blood lymphocyte counts in FACS analysis, we set out to further determine the impact of this lymphocyte depletion on immune infiltration in the CNS. To this end, we screened the cerebella of mice treated at the peak of EAE and ~ 60 days after EAE onset for CD3^+^ T cell and B220^+^ B cell infiltration by IHC. The tissue was collected 11.37 ± 0.18 days after treatment. To further differentiate infiltrate categories in the CNS, we defined three different types of lymphocyte infiltration (Fig. 3a–c). Loose perivascular infiltrates of CD3^+^ T cells in the absence of B220^+^ B cells were defined as non-B cell infiltrates, while the simultaneous presence of individual CD3^+^ T cells and B220^+^ B cells was characteristic of diffuse B cell infiltrates. B cell aggregates were defined as highly compact perivascular aggregation of stained lymphocytes containing at least one-third B220^+^ B cells. The percentage of perivascular infiltration per parenchymal area was determined for each category in acute and chronic EAE. As shown in Fig. 3, there was a significant overall reduction in immune cell infiltration in the cerebella of anti-mCD52-treated animals in the acute (*p* = 0.03) (Fig. 3d–f) and chronic EAE cohort (*p* = 0.002) (Fig. 3g–i). Strikingly, B cell aggregates were entirely absent in all of the *n* = 14 anti-mCD52-treated animals, while only *n* = 4 of 15 isotype control-treated mice were aggregate-free. Because of this complete depletion of B cell aggregates in the anti-CD52-treated group, we did not perform any further analysis on potential lymphoid neogenesis resulting from B cell aggregation in this study.

### Anti-mCD52 therapy does not have neuroprotective effects in MP4-induced EAE

**Fig. 4:**
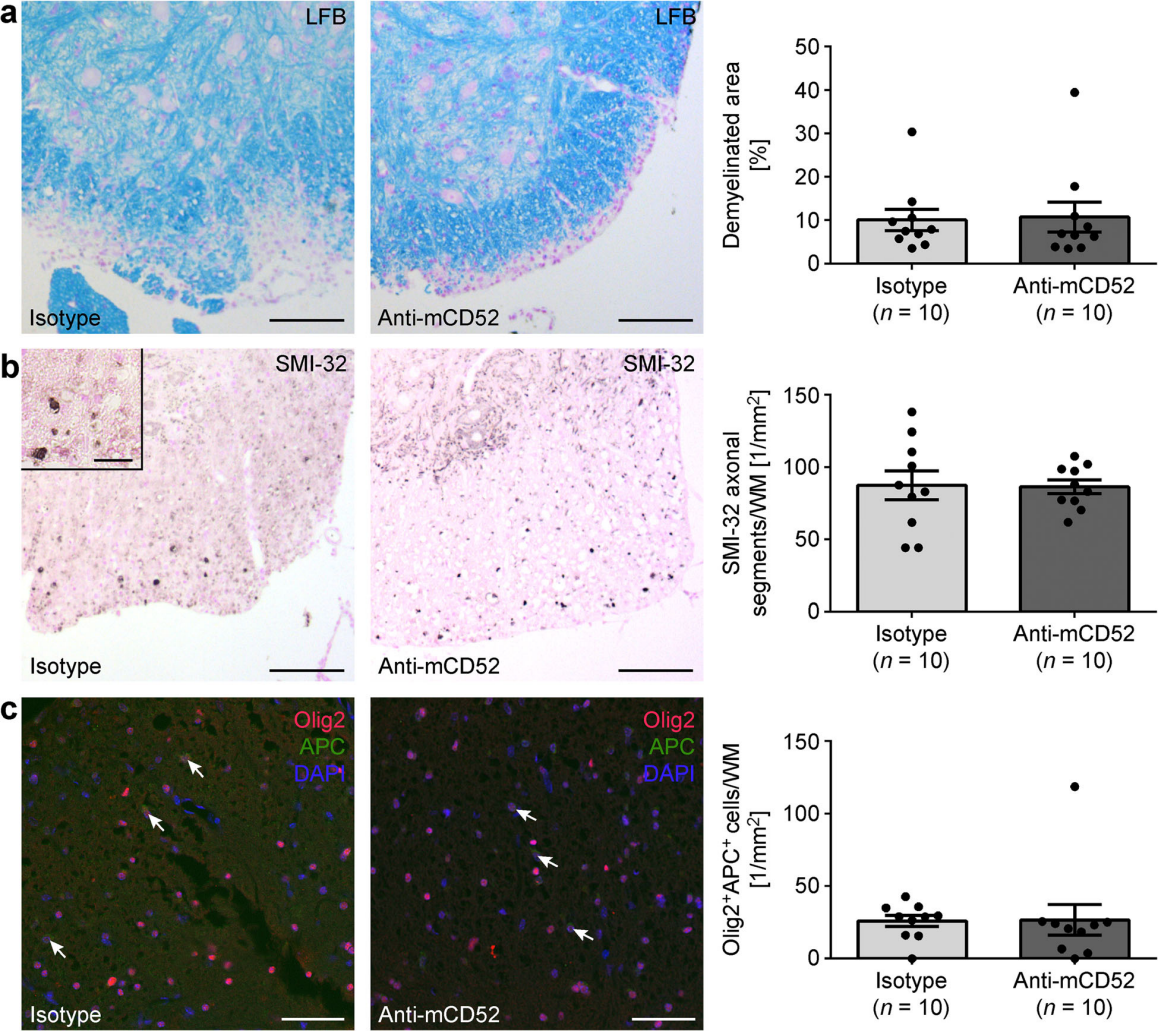
Detection of spinal cord demyelination, axonal damage, and remyelination in MP4-immunized mice. **a** Luxol Fast Blue staining for the detection of demyelination in spinal cord white matter. **b** IHC for SMI-32 for the detection of axonal damage in spinal cord white matter. **c** IHC for Olig2^+^APC^+^ oligodendrocytes in spinal cord white matter. Analysis was performed in mice that had received treatment in the chronic stage of EAE. Scale bars denote 100 μm. Graphs show means ± SEM

**Fig. 5:**
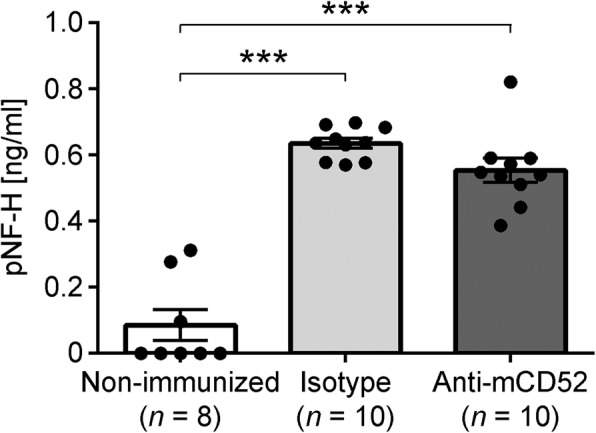
ELISA detection of pNF-H in MP4-immunized mice. The assay was performed on serum samples from non-immunized control mice as well as from isotype control- and anti-mCD52-treated MP4-immunized EAE mice that had received treatment ~ 60 days after EAE onset. Graphs show means ± SEM. ***p < 0.001, ANOVA

We have demonstrated that anti-mCD52 treatment significantly reduced both peripheral lymphocyte counts and CNS infiltration in MP4-induced EAE. Yet, there was no impact on clinical disease severity in the chronic stage of the disease. This lack of effect on late-stage EAE may be explained by the absence of primarily neuroprotective properties of the drug. We stained sections of the lumbar spinal cord of anti-mCD52- and isotype control-treated B6 mice of the chronic treatment cohort for demyelination using LFB (Fig. 4a) and for SMI-32^+^ damaged axons (Fig. 4b). Both the demyelinated area and the number of SMI-32^+^ axons were set in relation to the total white matter (WM) spinal cord area that was analyzed. We also determined the cell densities of Olig2^+^APC^+^ oligodendrocytes (Fig. 4c). The data show that the extent of demyelination (*p* = 0.74) and axonal damage (*p* = 0.97) as well as the cell densities of Olig2^+^APC^+^ oligodendrocytes (*p* = 0.07) were comparable between anti-mCD52- and isotype control-treated mice. Similar results were obtained when the cerebellum was analyzed (Additional file 3). To further corroborate these results, we performed pNF-H ELISA on serum samples from the same mice that were used for histology (Fig. 5). It has previously been proposed that the detection of pNF-H in CSF and blood of patients with neurodegenerative diseases provides information about the degree of axonal injury [26]. Indeed, pNF-H has been reported to be present in large amounts in CSF and blood following experimental spinal cord and brain injury in rats [27]. In addition, the detection of pNF-H has been associated with amyotrophic lateral sclerosis [28, 29], optic neuritis [30], and also with MS [31]. In our study, elevated levels of pNF-H were detectable in the sera of MP4-immunized compared to non-immunized control mice. However, there was no difference between anti-CD52- and isotype control-treated mice (Fig. 5).

**Fig. 6:**
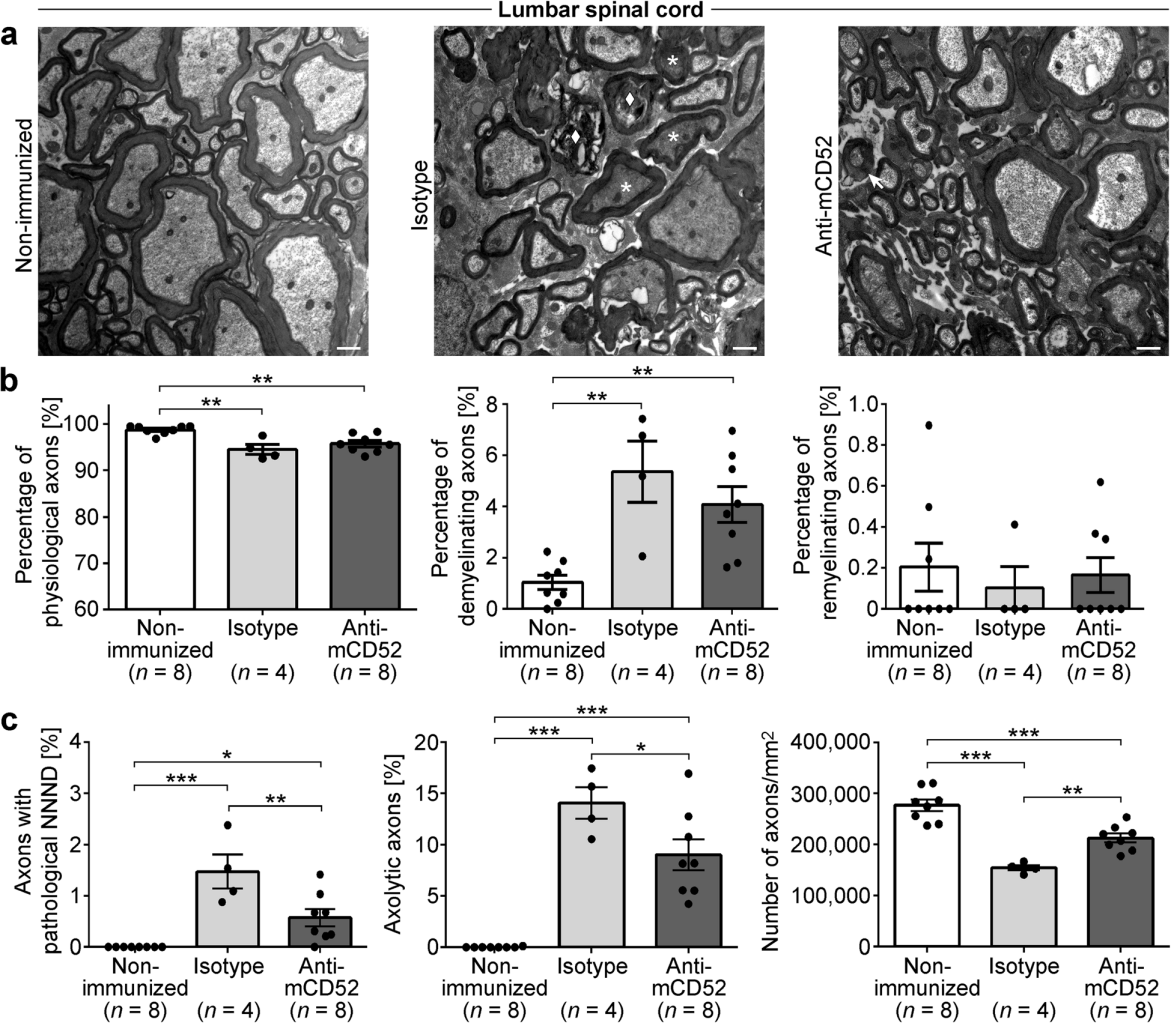
Electron microscopic analysis of demyelination and axonal damage in the spinal cord of MP4-immunized mice. **a**–**c** Representative electron microscopic images of the spinal cord of non-immunized control and in MP4-immunized mice that had received treatment with either isotype control or anti-mCD52 antibody ~ 60 days after EAE onset. Scale bars denote 1 μm. The arrow indicates a demyelinating axon. The asterisks represent axons with a decreased NNND, and the diamond shows an axolytic axon. **b** Quantification of the percentage of physiological and of de- and remyelinating nerve fibers in non-immunized control vs. MP4-immunized mice of the two treatment cohorts. **c** Quantification of axonal damage and loss in non-immunized control vs. MP4-immunized mice of the two treatment cohorts. Graphs show means ± SEM. *p < 0.05, **p < 0.01, ***p < 0.001, ANOVA

**Fig. 7:**
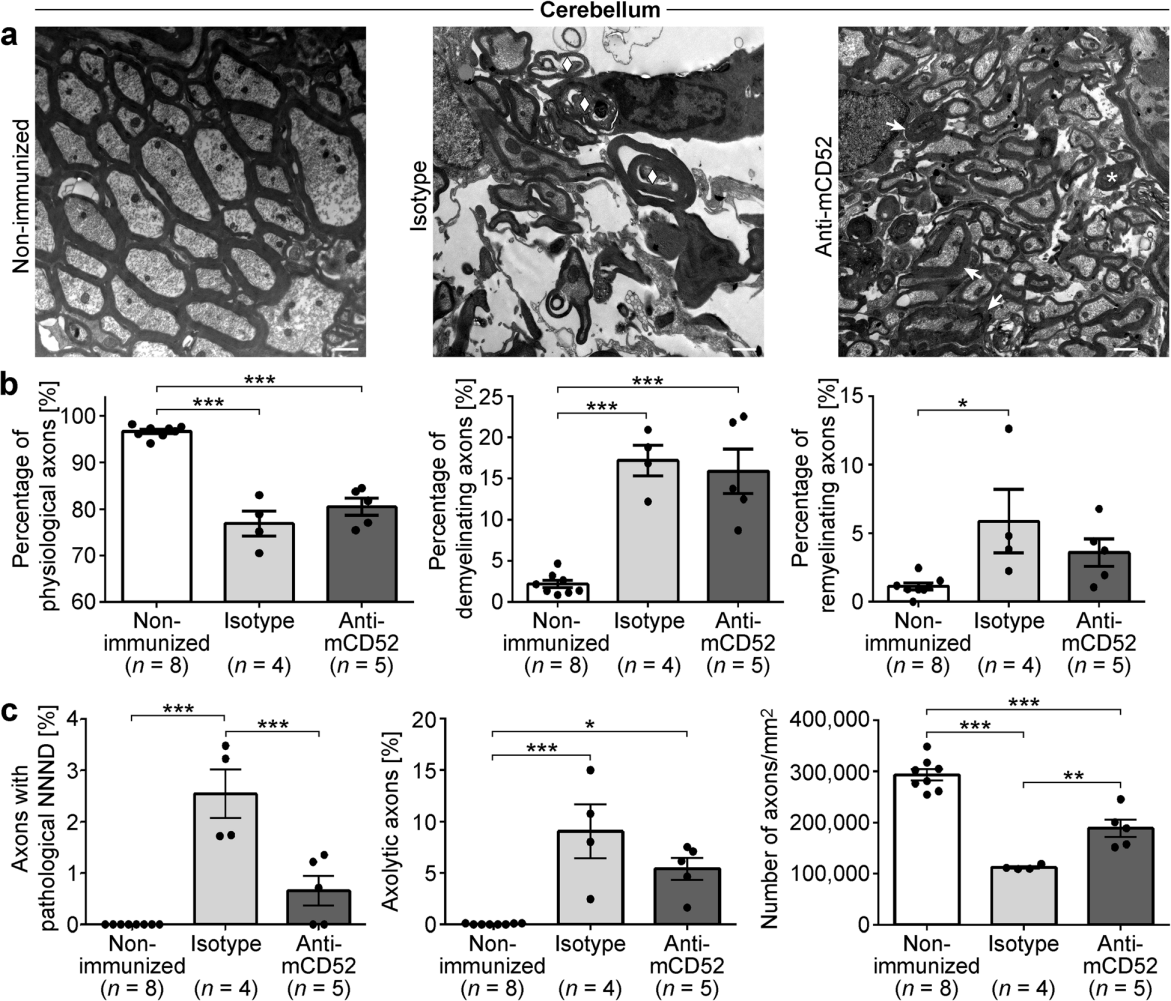
Electron microscopic analysis of demyelination and axonal damage in the cerebellum of MP4-immunized mice. **a**–**c** Representative electron microscopic images of the cerebellum of non-immunized control and MP4-immunized mice that had received treatment with either isotype control or anti-mCD52 antibody ~ 60 days after EAE onset. Scale bars denote 1 μm. The arrow indicates a demyelinating axon. The asterisk represents an axon with a decreased NNND, and the diamond shows an axolytic axon. **b** Quantification of the percentage of physiological and of de- and remyelinating nerve fibers in non-immunized control vs. MP4-immunized mice of the two treatment cohorts. **c** Quantification of axonal damage and loss in non-immunized control vs. MP4-immunized mice of the two treatment cohorts. Graphs show means ± SEM. *p < 0.05, **p < 0.01, ***p < 0.001, ANOVA

While light microscopic and serum analyses provide a first impression on neurodegeneration, ultrastructural analysis is needed for detailed quantification. To this end, we used electron microscopy to assess myelin pathology as well as beginning and irreversible axonal damage in both anti-mCD52- and isotype control-treated mice in the spinal cord (Fig. 6) and cerebellum (Fig. 7). In order to distinguish between de- and remyelinating nerve fibers, the g-ratio was used. This ratio describes the relationship of the myelin sheath to its respective nerve fiber. Analysis of *n* = 8 naïve mice allowed us to determine the physiological range of the g-ratio in both the spinal cord (0.40–0.86) and cerebellum (0.48–0.82). While beginning axonal damage can be assessed by the so-called nearest neighbor neurofilament distance (NNND) with a decreased NNND indicating pathology, irreversible axonal damage is reflected by axolysis and axonal loss. Figures 6 and 7 demonstrate that both anti-mCD52- and isotype control-treated mice displayed a significantly increased number of pathological nerve fibers both in terms of demyelination and axonal damage, which pertained both to the spinal cord and the cerebellum. Along these lines, the number of axons was significantly reduced in both EAE groups. While there was no difference in the extent of de- and remyelination comparing anti-mCD52- and isotype control-treated mice (Figs. 6b and 7b), anti-mCD52-treated displayed significantly decreased axonal pathology and axonal loss (Figs. 6c and 7c).

## Discussion

Alemtuzumab, a humanized anti-CD52 antibody, is a relatively new approved treatment option for MS with promising results in multiple clinical trials [32–35]. Yet, the mechanistic effects of the drug on different aspects of MS pathogenesis have remained subject to further research, including the impact of alemtuzumab on the B cell component of the disease. The most recent reports of treatment failure in individual patients with subsequent severe and possibly B cell-mediated relapses underline the importance of further research along these lines [36]. While longitudinal studies on the impact of alemtuzumab on peripheral and CNS B cells are difficult to perform in MS patients themselves, the EAE model offers the possibility of investigating long-term effects of anti-CD52 antibody treatment on both the autoimmune response and also on neurodegeneration and nueroregeneration.

In our model, anti-CD52 antibody treatment drastically reduced the numbers of circulating CD4^+^ T cells and B220^+^ B cells, while the titers of MP4-specific serum antibodies and the numbers of the corresponding plasmablast population in the blood remained unaffected. This was expected and well in line with previous studies in EAE [19–21] and in humans [12, 13, 15, 37] and could be explained by differences in the expression of CD52 on different lymphocyte subsets. In particular, low levels of CD52 on plasmablasts have been noted before [37].

Unlike previous studies that all relied on T cell-dependent EAE, we immunized mice with the MP4 molecule that induces both encephalitogenic T cells and B cells/antibodies. Using this model, we were able to show that anti-CD52 treatment did not only deplete circulating B cells, but also B cells that had formed aggregates in the CNS. Interestingly, treatment not only prevented the formation of aggregates, but also disrupted already existing aggregates in MP4-induced EAE. The clinical relevance of B cell aggregates in the brains of MS patients is still subject to debate, but an association with an earlier disease onset, progression, and death and with more severe cortical pathology is assumed [38, 39]. These results may support recently published data that found alemtuzumab to reduce the relapse rate in MS patients more efficiently than fingolimod, while being comparable to natalizumab [40]. Mechanistically, it is unclear whether the disruption of B cell aggregates in the brain after anti-CD52 antibody treatment is the result of direct B cell depletion or rather due to a lack of replenishment from circulating peripheral B cells. Natalizumab is a potent anti-VLA4-antibody that blocks the migration of lymphocytes through the blood-brain barrier (BBB) by inhibiting their binding to the cell adhesion molecules VCAM-1 and MAdCAM-1 on the endothelium. The resulting complete or partial disappearance of OCBs in the CSF of a group of RRMS patients treated with natalizumab [41, 42] indicates that constant supply from the periphery is necessary to maintain B cell aggregates if they are considered at least partially responsible for OCB development. We have previously shown that treatment with the S1P_1_ receptor agonist fingolimod, which inhibits lymphocyte egress from secondary lymphoid organs, prevented the development of B cell aggregates in the CNS in acute MP4-induced EAE, while it did not affect existing infiltrates in the chronic stage of the disease [43]. This rather suggests the independence of B cell aggregates, at least partially, from peripheral support. Yet, our data have also shown that the effect of fingolimod on B cells was much less pronounced than on T cells. Therefore, it is conceivable that there is still ongoing migration of autoreactive B cells into the CNS in the setting of fingolimod treatment, while natalizumab blocks B cell migration more effectively and alemtuzumab depletes the majority of migrating lymphocytes.

Any efficient depletion of lymphocytes in the CNS relies on the penetration of the antibody through the BBB, on sufficient binding to the target cell, and on the activation of antibody-dependent cellular and/or complement-dependent cytotoxicity (ADCC/CDC) in case of the currently available type I antibodies. This has been confirmed for alemtuzumab in vitro [44] and in vivo [17]*.* While in gadolinium-enhanced magnetic resonance imaging (MRI) studies of lesions in RR-MS patients the BBB has been described to be permeable for antibodies, and complement levels have been reported to be elevated in the CNS, the BBB in secondary progressive (SP-MS) and primary progressive (PP-MS) patients exhibits lower permeability. Indeed, a clinical trial comparing a combination of intrathecal and intravenous application of the anti-CD20 antibody rituximab to placebo in patients with low-inflammatory SP-MS was terminated early since it failed to reach the expected efficiency in the depletion of intrathecal B cells and axonal damage markers in the CSF [45]. Decreased CDC, lack of natural killer cells, and lower CSF flow were made responsible for the failure of the trial. These arguments may also apply to alemtuzumab treatment.

In order to prevent further or even reverse pre-existing neurological disabilities, the search for new treatment options for MS patients focuses not only on immune modulation, but also on neuroprotection [46, 47]. A post hoc subgroup analysis of patients in the CAMMS223 cohort with no clinical evidence of immunological disease activity before treatment and no clinical and radiological disease activity on-trial displayed an overall reduction of clinical disabilities [48]. This could not be explained by the suppression of inflammation due to the depletion of lymphocytes since ongoing neuroinflammation was not detectable in this subgroup. These findings raised hope that alemtuzumab may create an environment of “neuroprotective autoimmunity,” with reported higher levels of neurotrophins and oligotrophins resulting in disability reduction. Along these lines, Turner et al. [19] described the reversal of EAE scores in the MOG_35–55_, MOG_1–121_, and PLP_139–151_ model early after disease onset after anti-CD52 antibody treatment. Furthermore, they demonstrated neuroprotective effects of the drug in the spinal cord of MOG_35–55_-immunized mice during acute EAE, reporting a significant reduction in demyelinated white matter, axonal damage, as well as the preservation of axonal conductance. When we applied the anti-CD52 antibody at the peak of MP4-induced EAE, we observed a similar attenuation of clinical EAE. However, there was no effect on EAE scores when treatment was initiated in the chronic stage of the disease ~ 60 days after EAE onset, which had not been evaluated in the other EAE models before [19–21]. Further analysis revealed differential effects of the treatment on CNS degeneration in chronic MP4-immunized mice. In contrast to the results of Turner et al., we did not observe any difference between isotype- and anti-CD52 antibody-treated mice in the light microscopic analysis of demyelination and SMI-32^+^ axons, indicating axonal damage in the lumbar spinal cord and cerebellum of MP4-immunized mice. These results were underlined by similar levels of pNF-H in the sera obtained from the two mouse groups. Furthermore, we did not observe ongoing remyelination when staining for Olig2^+^APC^+^ oligodendrocytes or when performing ultrastructural electron microscopic analysis. Yet, on the ultrastructural level, the numbers of damaged axons were significantly decreased after anti-CD52 treatment. Indeed, electron microscopy allows the precise evaluation of CNS pathology on the level of individual nerve fibers and is therefore much more sensitive than the light microscopic techniques. These beneficial effects on CNS histopathology are likely to the result of reduced CNS inflammation and potentially the disruption of B cell aggregates, which have been described to secrete soluble toxic molecules into the adjacent tissue inducing CNS pathology [39]. This assumption is supported by a 6-year follow-up clinical trial that displayed sustained reduction of the relapse rate, however without improvement of clinical disabilities in a more selective group of alemtuzumab-treated patients with poor prognostic indicators such as high relapse rate, active disease on cMRI scans, and rapidly accumulating disability [34].

## Conclusion

This study underlines the potency of anti-CD52 antibody treatment to deplete T cells and B cells in the context of EAE. The novel aspect of this study is the demonstration of a treatment effect on B cell aggregates in the CNS. It will be subject to future studies to determine whether alemtuzumab is equally effective in depleting B cell aggregates in MS patients and whether such depletion is actually associated with clinical benefit. On the one hand, suitable imaging tools to detect B cell aggregate formation in the brains of MS patients would help to address this question. Until such tools may eventually be available, the development of blood- and/or CSF-based biomarkers to measure autoantigen-specific B cell activity may be useful to predict and monitor the effect of alemtuzumab on the B cell compartment in patients and to correlate such an effect with clinical outcomes. On the other hand, while B cell aggregate formation is a characteristic feature of the MP4 model, the actual existence and relevance of these structures in the MS brain is still heavily debated.

Eventually, the permeability of alemtuzumab through the BBB and its mode of action in the CNS itself needs to be further investigated. It may also be worthwhile to consider a type II anti-CD52 antibody, which is supposed to be less dependent on CDC and ADCC by inducing apoptosis directly.

## Additional files

Additional file 1: Gating strategy for flow cytometric analysis of viable CD19^+^ B cells and CD4^+^ T cells in the blood. (TIF 1297 kb)

Additional file 2: Comprehensive disease course of mice treated with anti-mCD52 antibody in the acute or chronic stage of EAE, respectively. (TIF 143 kb)

Additional file 3: Detection of cerebellar demyelination, axonal damage, and remyelination in MP4-immunized mice. (a) Luxol Fast Blue staining for the detection of demyelination in the cerebellum. (b) IHC for SMI-32 for the detection of axonal damage in the cerebellum. (c) IHC for Olig2^+^APC^+^ oligodendrocytes in the cerebellum. Analysis was performed in mice that had received treatment in the chronic stage of EAE. The scale bar denotes 50 μm in (a) and (b) and 100 μm in (c). (TIF 17531 kb)

## Abbreviations

ADCC: Antibody-dependent cellular cytotoxicity
B6: C57BL/6
BBB: Blood-brain barrier
CACO: Cacodylate
CDC: Complement-dependent cytotoxicity
CFA: Complete Freund’s adjuvant
CNS: Central nervous system
CSF: Cerebrospinal fluid
DAB: Diaminobenzidine
EAE: Experimental autoimmune encephalomyelitis
GA: Glutaraldehyde
IHC: Immunohistochemistry
IL-4: Interleukin-4
LFB: Luxol Fast Blue
MBP: Myelin basic protein
MOG: Myelin oligodendrocyte glycoprotein
MP: Milk powder
MP4: MBP-PLP fusion protein
MRI: Magnetic resonance imaging
MS: Multiple sclerosis
NGS: Normal goat serum
NNND: Nearest neighbor neurofilament distance
OCB: Oligoclonal bands
PBS: Phosphate-buffered saline
PFA: Paraformaldehyde
PLP: Proteolipid protein
pNF-H: Phosphorylated neurofilament heavy
PP-MS: Primary progressive MS
RR-MS: Relapsing-remitting MS
SEM: Standard error of the mean
SP-MS: Secondary progressive MS
WM: White matter
