# Supplementary figures and images for: Anti-CD52 antibody treatment depletes B cell aggregates in the central nervous system in a mouse model of multiple sclerosis
Source: J Neuroinflammation. 2018 Aug 11;15:225. doi: 10.1186/s12974-018-1263-9 (PMC6086993; doi:10.1186/s12974-018-1263-9)

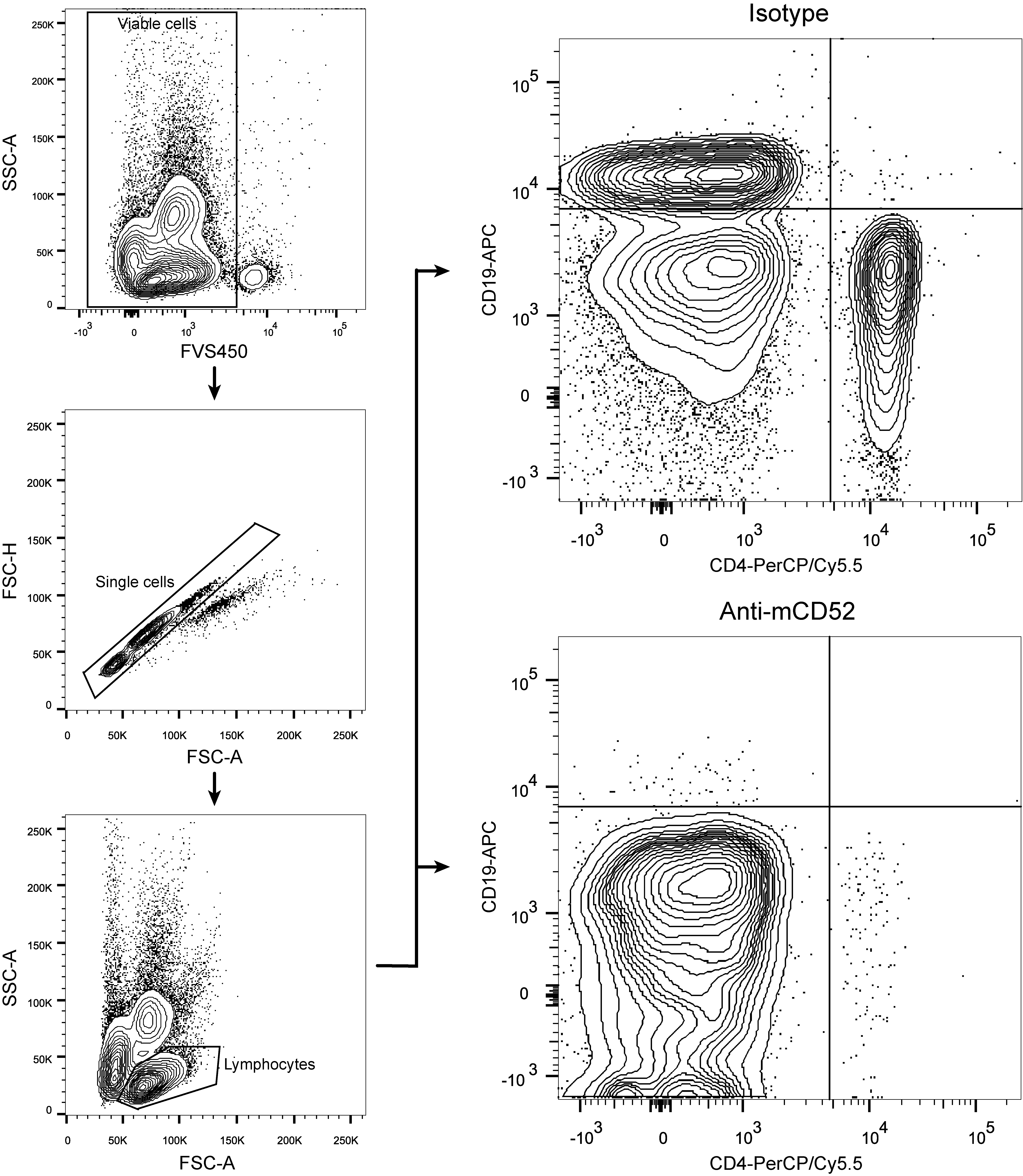

Supplement: Supplementary file 1 — Gating strategy for flow cytometric analysis of viable CD19+ B cells and CD4+ T cells in the blood. (TIF 1297 kb) [file 12974_2018_1263_MOESM1_ESM.tif]

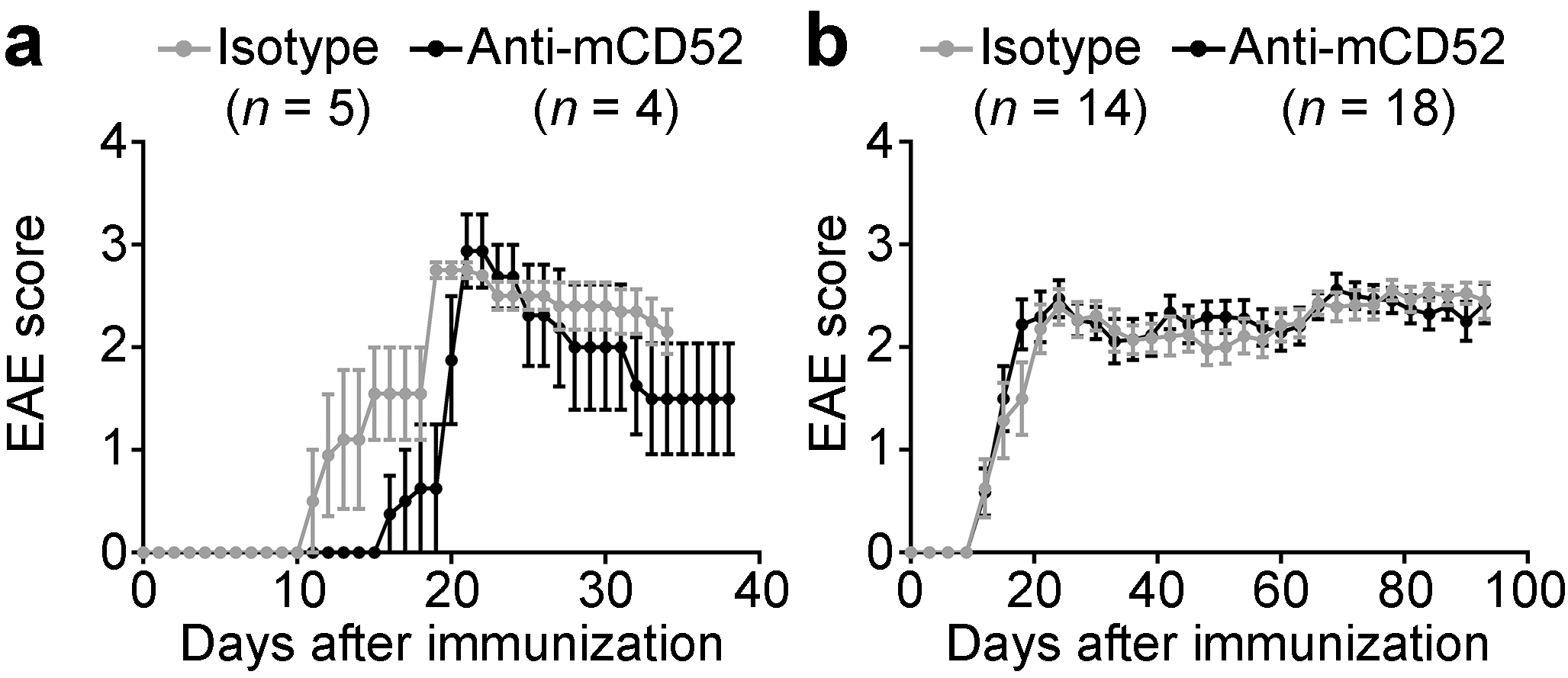

Supplement: Supplementary file 2 — Comprehensive disease course of mice treated with anti-mCD52 antibody in the acute or chronic stage of EAE, respectively. (TIF 143 kb) [file 12974_2018_1263_MOESM2_ESM.tif]

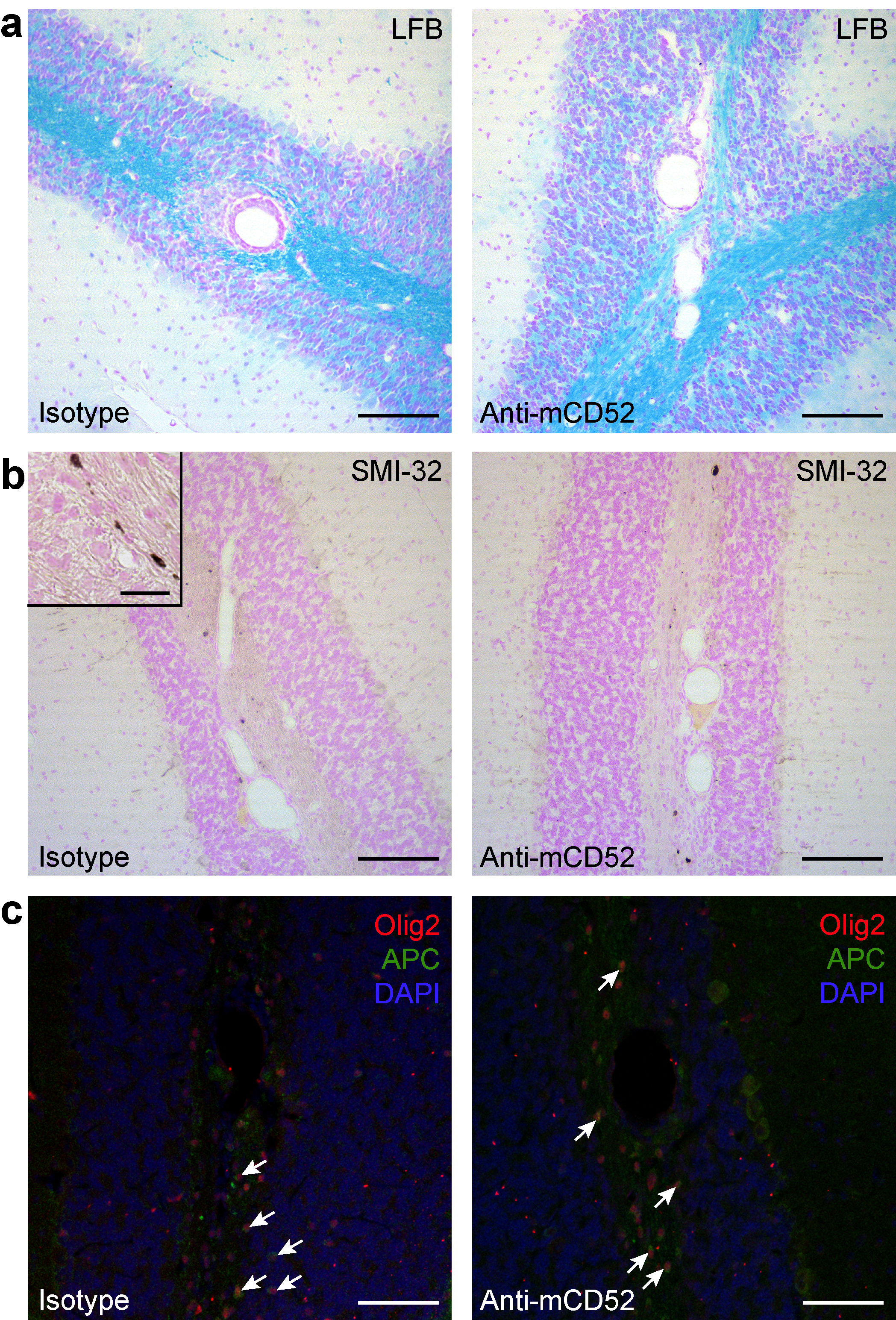

Supplement: Supplementary file 3 — Detection of cerebellar demyelination, axonal damage, and remyelination in MP4-immunized mice. (a) Luxol Fast Blue staining for the detection of demyelination in the cerebellum. (b) IHC for SMI-32 for the detection of axonal damage in the cerebellum. (c) IHC for Olig2+APC+ oligodendrocytes in the cerebellum. Analysis was performed in mice that had received treatment in the chronic stage of EAE. The scale bar denotes 50 μm in (a) and (b) and 100 μm in (c). (TIF 17531 kb) [file 12974_2018_1263_MOESM3_ESM.tif]
